# Biochemical characterization of the Eya and PP2A-B55α interaction

**DOI:** 10.1016/j.jbc.2024.107408

**Published:** 2024-05-23

**Authors:** Christopher Alderman, Ryan Anderson, Lingdi Zhang, Connor J. Hughes, Xueni Li, Chris Ebmeier, Marisa E. Wagley, Natalie G. Ahn, Heide L. Ford, Rui Zhao

**Affiliations:** 1 Department of Biochemistry and Molecular Genetics, University of Colorado Anschutz Medical Campus, Aurora, Colorado, USA; 2 Medical Scientist Training Program, University of Colorado Anschutz Medical Campus, Aurora, Colorado, USA; 3 Molecular Biology Program, University of Colorado Anschutz Medical Campus, Aurora, Colorado, USA; 4 Department of Pharmacology, University of Colorado Anschutz Medical Campus, Aurora, Colorado, USA; 5 Department of Biochemistry, University of Colorado-Boulder, Boulder, Colorado, USA

**Keywords:** Eya, protein phosphatase 2A (PP2A), Myc, phosphatase, cancer

## Abstract

The eyes absent (Eya) proteins were first identified as co-activators of the six homeobox family of transcription factors and are critical in embryonic development. These proteins are also re-expressed in cancers after development is complete, where they drive tumor progression. We have previously shown that the Eya3 N-terminal domain (NTD) contains Ser/Thr phosphatase activity through an interaction with the protein phosphatase 2A (PP2A)-B55α holoenzyme and that this interaction increases the half-life of Myc through pT58 dephosphorylation. Here, we showed that Eya3 directly interacted with the NTD of Myc, recruiting PP2A-B55α to Myc. We also showed that Eya3 increased the Ser/Thr phosphatase activity of PP2A-B55α but not PP2A-B56α. Furthermore, we demonstrated that the NTD (∼250 amino acids) of Eya3 was completely disordered, and it used a 38-residue segment to interact with B55α. In addition, knockdown and phosphoproteomic analyses demonstrated that Eya3 and B55α affected highly similar phosphosite motifs with a preference for Ser/Thr followed by Pro, consistent with Eya3’s apparent Ser/Thr phosphatase activity being mediated through its interaction with PP2A-B55α. Intriguingly, mutating this Pro to other amino acids in a Myc peptide dramatically increased dephosphorylation by PP2A. Not surprisingly, Myc^P59A^, a naturally occurring mutation hotspot in several cancers, enhanced Eya3-PP2A-B55α–mediated dephosphorylation of pT58 on Myc, leading to increased Myc stability and cell proliferation, underscoring the critical role of this phosphosite in regulating Myc stability.

The mammalian family of sine oculis homeobox (Six) transcription factors is composed of six members (Six1–6) that are homologs of the protein sine oculis in *Drosophila* (1). This family of proteins relies on the eyes absent (Eya) family of co-activators, consisting of Eya1–4 in mammals, to activate transcription (2, 3, 4). We will use the mouse nomenclature such as Six and Eya to represent proteins from both human and mouse given their extensive sequence homologies, and we will detail the species of proteins used in specific experiments in the Experimental procedures section. Six family members interact with Eya proteins through the Six domain of Six and Eya domain (ED) of Eya. Together, Six and Eya families play important roles in epithelial to mesenchymal transition, cell proliferation, and differentiation (2, 3, 5) during embryonic development. Six and Eya families typically lose expression in mature tissues; however, when they are aberrantly expressed in postdevelopment tissues, the Six-Eya transcriptional complex can drive oncogenic processes by reactivating developmental pathways (5, 6, 7, 8, 9, 10, 11). Six-Eya has been shown to play a critical role in tumor progression in multiple cancer types including those of the breast, cervix, ovaries, liver, and gastrointestinal tract (12, 13, 14, 15, 16, 17, 18, 19, 20, 21, 22, 23, 24).

In addition to their role as transcriptional co-activators, the C-terminal ED of Eya members contains haloacid dehydrogenase-family Tyr phosphatase activity involved in regulating cellular functions including transcriptional activation, DNA damage response, angiogenesis, and cell motility (2, 3, 19, 25, 26, 27, 28, 29, 30). The conserved transactivation N-terminal domain (ED2/NTD) is Pro/Ser/Thr rich and contains Ser/Thr phosphatase activity (12, 31). The apparent Thr phosphatase activity of Eya3 is more pronounced than its Ser phosphatase activity and contributes to innate immunity (32, 33). Our previous work has shown that the apparent Ser/Thr phosphatase activity of Eya3 derives from an interaction with protein phosphatase 2A (PP2A)-B55α (12).

PP2A is the primary Ser/Thr phosphatase in the cell (6) that dephosphorylates signaling components involved in metabolism, cell cycle progression, DNA replication, translation, cell migration, oncogenic transformation, apoptosis, and stress response (34). The PP2A holoenzyme is a trimer with A, B, and C subunits functioning as the scaffold, regulatory, and catalytic components, respectively (6). The A subunit has homologous Aα and Aβ isoforms, while the C subunit has homologous Cα and Cβ isoforms (6). The A and C subunits form the core enzyme and require the B subunit to achieve full activity, substrate specificity, and localization of the holoenzyme. There are at least 26 B isoforms that belong to four subfamilies allowing for nuanced regulation of the enzymatic core (35).

PP2A plays an integral role in the regulation of Myc stability through modulating Myc’s phosphorylation status (36, 37). Myc is a key oncogene that is overexpressed in 65% of human cancers (38) and drives several oncogenic mechanisms including cell growth, chromatin modification, miRNA expression, transcription regulation, and biomass accumulation (39). The stability of Myc is regulated by the dynamic phosphorylation and dephosphorylation of the T58 and S62 residues. Briefly, ERK phosphorylates S62, followed by GSK3β phosphorylation of T58 (40). Pin1 causes cis-trans isomerization of P63, facilitating pS62 dephosphorylation by PP2A-B56α and subsequent degradation of pT58 Myc through the proteasome pathway (36, 37). Thus, PP2A has been viewed as a tumor suppressor, which is supported by data showing that PP2A inhibitors, PP2A mutants, and loss of PP2A activation contribute to oncogenesis (6). On the other hand, some studies have revealed that PP2A can work as an oncogene by inhibiting apoptosis through Bcl-2 and P53 dephosphorylation (41), suggesting that PP2A’s role in cancer depends on the cellular context.

We have shown that Eya3-PP2A-B55α dephosphorylates pT58 and increases Myc stability (12), and both Eya3 and B55α promoted metastasis in a mouse model of triple-negative breast cancer (12). These observations revealed Eya3 as a new regulator of PP2A-B55α activity and Myc stability. In this study, we conducted further biochemical characterization of the Eya3 and PP2A-B55α interaction and revealed that Eya proteins use a disordered segment consisting of 38 amino acids to interact with PP2A-B55α.

## Results

### The C-terminal domain of Eya3 directly interacts with the NTD of Myc

**Figure 1:**
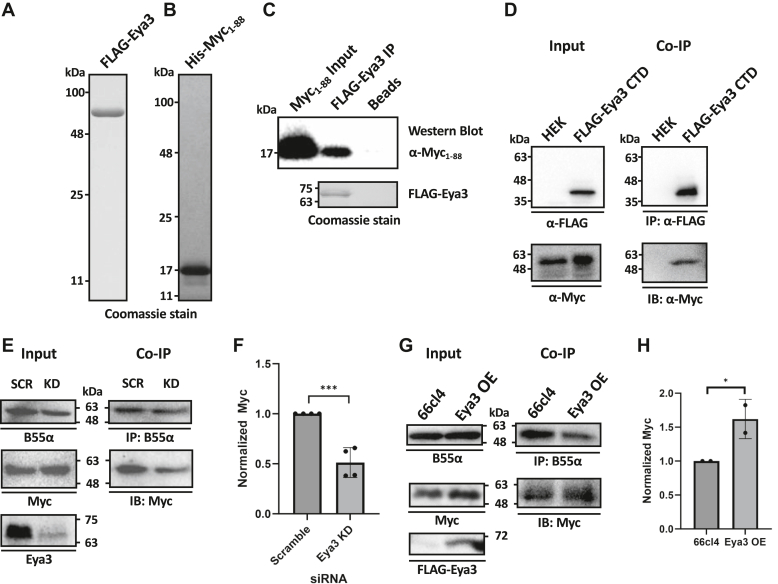
**Eya3 directly interacts with Myc.***A*, Coomassie stain of FLAG-Eya3 overexpressed and purified from *E. coli*. *B*, Coomassie stain of His-Myc_1-88_ overexpressed and purified from *E. coli*. *C*, purified FLAG-Eya3 and His-Myc_1-88_ were incubated, immunoprecipitated using an α-FLAG antibody, followed by probing of His-Myc_1-88_ with an α-Myc antibody in Western blot. The amount of FLAG-Eya3 used in immunoprecipitation was shown with Coomassie stain under the Western Blot. *D*, co-IP of FLAG-Eya3 CTD (residues 241–526) from HEK cells, followed by α-FLAG and α-Myc Western blot showed that the Eya3 CTD interacts with endogenous Myc. *E*, co-IP using an α-B55α antibody probed with α-B55α and α-Myc antibodies in 66cl4 cells with shRNA scramble control and α-Eya3 shRNA showed that Eya3 KD led to reduced association between B55α and Myc. *F*, quantification of data from panel *E*. *G*, co-IP using an α-B55α antibody probed with α-B55α and α-Myc antibodies in 66cl4 cells with and without FLAG-Eya3 overexpression (OE) showed that Eya3 OE led to increased association between B55α and Myc. *H*, quantification of data from panel *G*. ∗*p* < *0*.*05*, ∗∗∗*p* < 0.001 from four biological replicates in panel *F* and two biological replicates in panel *H*. Statistical analysis was done using an Fmax test followed by a two-tailed, homoscedastic *t* test. Error bars represent SD. CTD, C-terminal domain; Eya, eyes absent; IP, immunoprecipitation; KD, knockdown.

We have previously shown that Eya3 directly interacts with the B55α subunit of PP2A (12). To evaluate if Eya3 also directly interacts with Myc, we expressed and purified FLAG-Eya3 (Eya3) and His-Myc NTD (residues 1–88, Myc_1–88_) from *E. coli* (Fig. 1, *A* and *B*). Since full-length Myc is difficult to express and purify with *E. coli*, we started by looking at the interaction of Eya3 with Myc_1–88_ which contains key phosphorylation sites pT58 and pS62 and has been previously expressed and purified (42). Immunoprecipitation (IP) of FLAG-tagged Eya3 (FLAG-Eya3) showed that it interacted with Myc_1–88_ (Fig. 1*C*). We also demonstrated that the C-terminal domain (CTD) of Eya3 (residues 241–526) interacted with Myc using co-IP experiments from HEK293FT (HEK) cells transfected with FLAG-Eya3 CTD (Fig. 1*D*). We next co-immunoprecipitated B55α in mouse TNBC 66cl4 cells with Eya3 knockdown (KD) or containing a scrambled shRNA control. In the Eya3 KD cells, we observed a decrease in the interaction between B55α and Myc compared to the scrambled control (Fig. 1, *E* and *F*). We also found that overexpression of Eya3 increased the interaction between B55 and Myc through co-IP of endogenous B55α and Myc (Fig. 1, *G* and *H*). These data indicate that Eya3 enhances recruitment of B55α to Myc in cells.

### Eya3 specifically enhances PP2A-B55α activity

**Figure 2:**
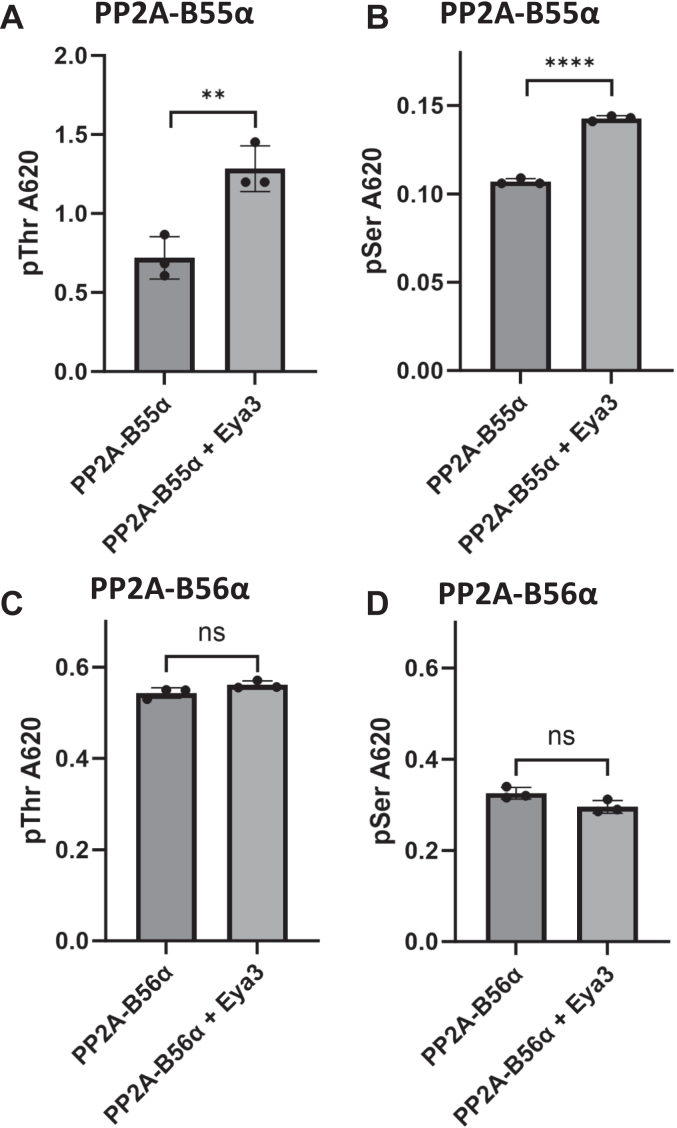
**Eya3 increases the phosphatase activity of PP2A-B55α but has no effect on the phosphatase activity of PP2A-B56α.***A*, PP2A-B55α phosphatase activity with the KRpTIRR peptide substrate in the presence and absence of FLAG-mouse Eya3 (mEya3) purified from *E. coli*. *B*, PP2A-B55α phosphatase activity with KRpSIRR substrate in the presence and absence of FLAG-mEya3. *C*, PP2A-B56α phosphatase activity with the KRpTIRR substrate in the presence and absence of FLAG-mEya3. *D*, PP2A-B56α phosphatase activity with the KRpSIRR substrate in the presence and absence of FLAG-mEya3. All phosphatase activities were determined using a malachite green assay with absorbance measured at 620 nm (*A*_620_). All statistical analysis was done on three biological replicates using an Fmax test followed by a two-tailed, homoscedastic *t* test. ∗∗*p* < 0.01, ∗∗∗∗*p* < 0.0001. Error bars represent the SD. Eya, eyes absent; PP2A, protein phosphatase 2A.

We next asked how Eya3 affects the activity of PP2A-B55α. We expressed and purified Eya3 and PP2A Aα from *E. coli*, and B55α and Cα from insect cells (Fig. S1) and assembled the PP2A-B55α holoenzymes. We analyzed the phosphatase activity of the holoenzymes using pThr (KRpTIRR) or pSer (KRpSIRR) generic peptide substrates in the presence or absence of mouse Eya3 purified from *E. coli* in malachite green assays. These assays showed that Eya3 increased the phosphatase activity of PP2A-B55α on pThr and pSer substrates (Fig. 2, *A* and *B*). We performed similar experiments with PP2A-B56α and demonstrated that Eya3 had no effect on the activity of PP2A-B56α (Fig. 2, *C* and *D*), consistent with our observation that Eya3 interacts with the B55α but not B56 subunit (12). These data also showed that both PP2A-B55α and B56α dephosphorylated pThr better than pSer, as previously reported (43), and Eya3 did not change this preference (Fig. 2).

### Eya3 uses a short segment in its disordered NTD to interact with B55α

**Figure 3:**
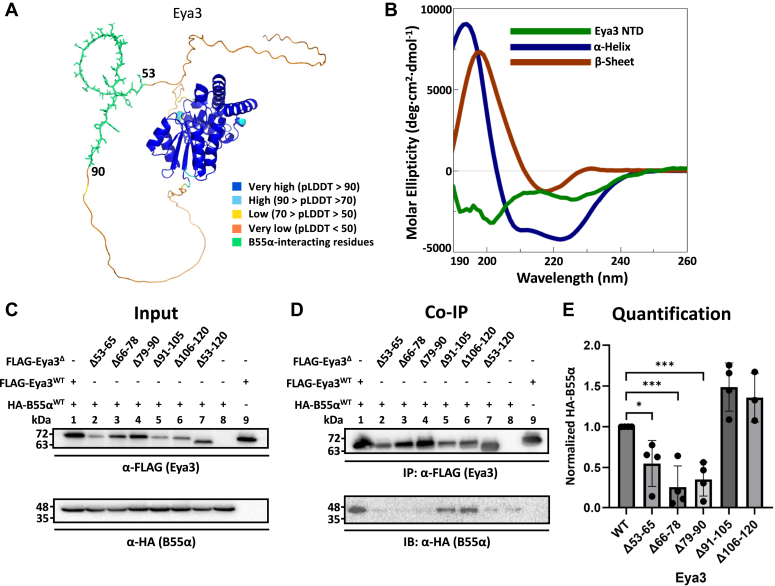
**Eya3 uses a short, intrinsically disordered segment within its NTD to interact with B55α.***A*, AlphaFold (45) prediction of Eya3. Very high confidence (predicted local distance difference test (pLDDT) > 90) was *blue*, high confidence (90 > pLDDT > 70) was *light blue*, low confidence (70 > pLDDT > 50) was *yellow*, and very low confidence (pLDDT <50) was *orange*. Residues (53–90) shown to bind B55α in panels *C*–*E* were shown in *green*. *B*, circular dichroism of purified Eya3 NTD (residues 1–287) confirmed it was disordered. *C*–*E*, FLAG-Eya3 with serial deletions within residues 53 to 120 and HA-B55α were cotransfected into HEK cells. FLAG co-IP followed by α-FLAG and α-HA probing in Western blot showed residues 53 to 90 were important for interacting with B55α. The ratio of HA-B55α to Flag-Eya3 in co-IPs were quantified in panel *C* with the ratio of WT B55a to Flag-Eya3 normalized to 1. *p* values were calculated from three biological replicates using a single-factor ANOVA, followed by Tukey’s test and the Fmax test, then a homoscedastic *t* test with the Bonferroni correction. ∗*p* < 0.05, ∗∗∗*p* < 0.001. Error bars represented the SD. Eya, eyes absent; IP, immpunoprecipitation; NTD, N-terminal domain.

We have previously shown that Eya3 interacts with B55α through its NTD (12). We next performed secondary structure prediction with JPred4 (44) and tertiary structure prediction with AlphaFold (45), which both predicted the entire Eya3 NTD to be disordered (Figs. S2*A* and 3*A*). We then performed circular dichroism analyses of recombinant Eya3 NTD and found that there were no secondary structures (Fig. 3*B*), consistent with the secondary and tertiary structure predictions.

Since the Eya3 NTD is intrinsically disordered, we were able to make serial deletions in the NTD without disrupting proper secondary or tertiary structure formation. Previous studies by the Nagata lab showed that Eya3 amino acids 53 to 120 are responsible for its apparent Ser/Thr phosphatase activity (31). We generated smaller serial deletions within the 53 to 120 region of Eya3. We cotransfected HEK cells with FLAG-Eya3 containing serial deletions in its NTD and HA-tagged B55α (HA-B55α). We then performed co-IP experiments of FLAG-Eya3 and detected HA-B55α. These experiments demonstrated that residues 53 to 90 of Eya3 interacted with B55α (Fig. 3, *C*–*E*). We also found that these deletions did not affect the cellular localization of FLAG-Eya3 (Fig. S3).

**Figure 4:**
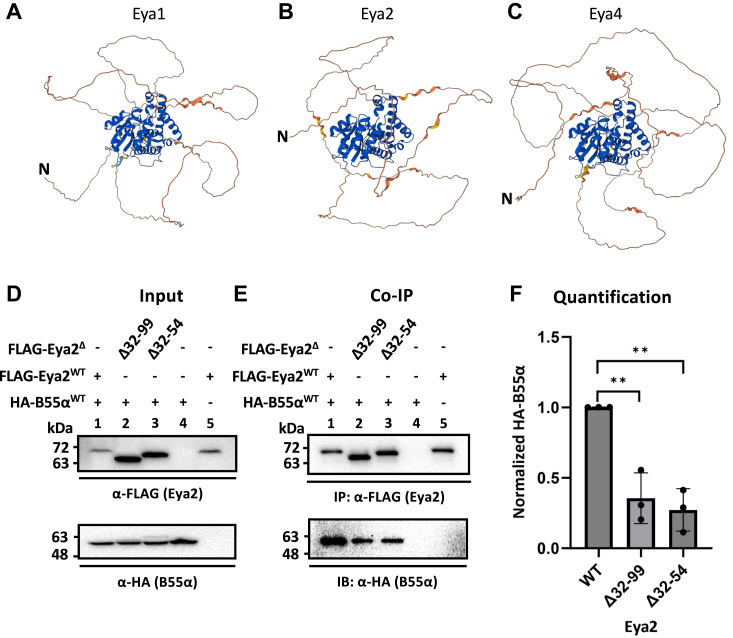
**Other Eya family members such as Eya2 also use a disordered segment in their NTD to interact with B55α.***A*–*C*, AlphaFold prediction of Eya1, 2, and 4 indicated they all contained a disordered NTD. Very high confidence (pLDDT >90) was *blue*, high confidence (90 > pLDDT > 70) was *light blue*, low confidence (70 > pLDDT > 50) was *yellow*, and very low confidence (pLDDT <50) was *orange*. The N termini of the proteins were marked by an “N”. *D*–*F*, FLAG-Eya2 NTD deletions and HA-B55α were cotransfected into HEK cells. FLAG co-IP followed by α-FLAG and α-HA probing in Western blot demonstrated that Eya2 32–54 were important for its interaction with B55α. *p* values were calculated from three biological replicates using a single-factor ANOVA, followed by Tukey’s test and the Fmax test, then a homoscedastic *t* test with the Bonferroni correction. ∗∗*p* < 0.01. Error bars represented the SD. Eya, eyes absent; NTD, N-terminal domain.

We next evaluated if other Eyas also use a disordered segment in their NTDs to interact with B55α. Both secondary structure and AlphaFold (45) predictions indicated that the NTDs of all Eyas were intrinsically disordered (Figs. 4, *A*–*C* and S2, *A*–*D*). Although we could not express and purify the NTD of Eya1, Eya2, or Eya4 to experimentally confirm these predictions, we decided to test if the equivalent segment in the NTD of the other Eya family members such as Eya2 was also important for its interaction with B55α. Sequence alignment (46) between Eya2 and Eya3 indicated that Eya2 residues 32 to 99 correspond to Eya3 residues 54 to 120 (Fig. S4, Eya3 residue 53 was not aligned with any residue in Eya2). We generated an Eya2 Δ32 to 99 and a smaller deletion Δ32 to 54 and cotransfected either one with HA-B55α in HEK cells. We next performed co-IP experiments of FLAG-Eya2 and probed HA-B55α. These experiments showed that deletion of residues 32 to 54, and 32 to 99, indeed significantly reduce the interaction between Eya2 and B55α, suggesting that all Eya family members use a disordered segment in its NTD to interact with B55α (Fig. 4, *D*–*F*).

### Eya3 interacts with B55α differently from pTau

**Figure 5:**
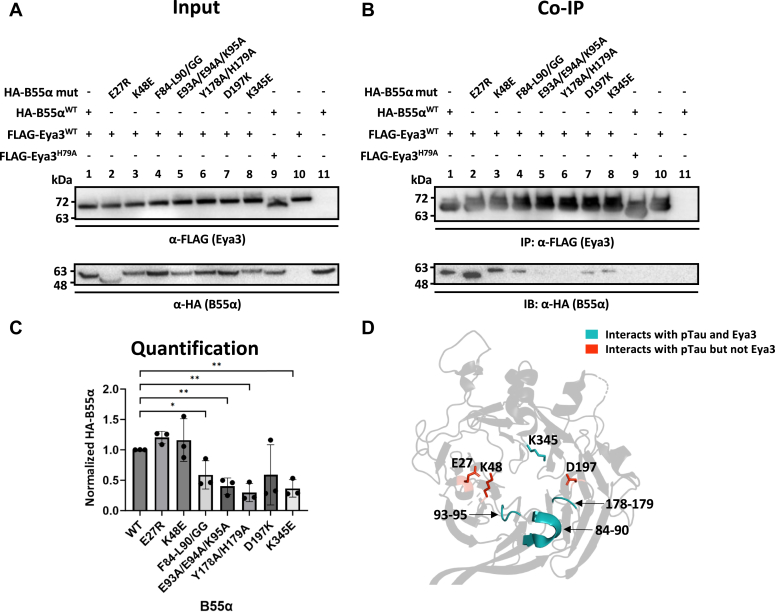
**B55α interacts with Eya3 differently than its interactions with substrates.***A*–*C*, FLAG-Eya3 and HA-B55α carrying various mutations were cotransfected into HEK cells. FLAG co-IP was performed followed by α-FLAG and α-HA probing in Western blot. Lanes 10 and 11 were transfected with Eya3 or B55α alone as negative controls. Lane 1 was transfected with both WT Eya3 and B55α as a positive control, and lane 9 was transfected with WT B55α and Eya3^H79A^ mutant which is known to disrupt its interaction with B55α (12). The ratio of HA-B55α to Flag-Eya3 in co-IPs were quantified in panel *C* with the ratio of WT B55a to Flag-Eya3 normalized to 1. *p* values were calculated from three biological replicates using a single-factor ANOVA, followed by Tukey’s test and the Fmax test, then a homoscedastic *t* test with the Bonferroni correction. ∗*p* < 0.05, ∗∗*p* < 0.01. Error bars represented the SD. *D*, the B55α structure (gray, PDB ID: 3DW8) with residues that interacted with pTau (47) in *sticks* for single amino acids and in *colored ribbon* for a range of amino acids (such as residues 84–90). *Teal* indicated residues that interact with both pTau and Eya3. *Red* indicated residues that interacted with pTau but did not interact with Eya3. Eya, eyes absent.

Previous Ala scanning and binding experiments have defined a largely acidic phosphatase substrate-binding pocket (residues E27, K48, F84-L90, E93, E94, K95, Y178, H179, D197, and K345) on the surface of B55α that interacts with pTau, and many of these residues also bind other PP2A-B55α substrates such as p107 and pRb (47, 48). To evaluate if these residues contribute to the interaction between B55α and Eya3, we cotransfected HEK cells with FLAG-Eya3 and HA-B55α carrying mutations of the above residues: E27R, K48E, F84-L90/GG, E93A/E94A/K95A, Y178A/H179A, D197K, and K345E (47). We performed co-IP experiments of FLAG-Eya3 and detected associated HA-B55α. We found a subset of mutations, including F84-L90/GG, E93A/E94A/K95A, Y178A/H179A, and K345E, affect the B55α and Eya3 interaction (Fig. 5, *A*–*C*). We mapped these residues on the crystal structure of B55α (47) and depicted residues interacting with both pTau and Eya3 in teal, and residues interacting with pTau but not Eya3 in red (Fig. 5*D*). Note that we did not know if single amino acid mutations in the F84-L90/GG, E93A/E94A/K95A, and Y178A/H179A mutants would disrupt Eya3 interaction and the actual number of residues involved in Eya3 binding might be less than indicated in Figure 5*D*. Taken together, our data suggest that the Eya3-binding surface of B55α is different from its acidic substrate-binding pocket with partial overlap.

### Eya3 and PP2A-B55α share similar phosphosite motif preferences

**Figure 6:**
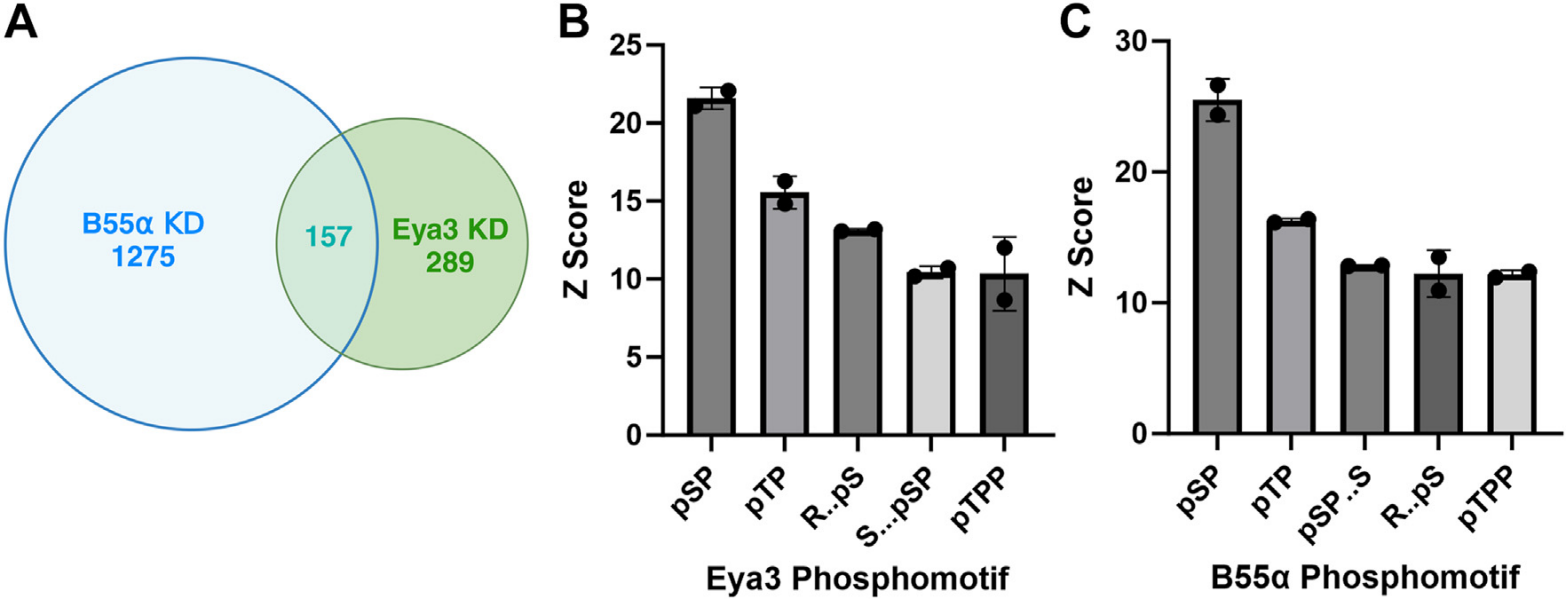
**Eya3 and B55α KD affect the same phosphosite motifs.** HEK cells with Eya3 or B55a KD were subjected to phosphoproteomic analyses. *A*, number of phosphosites significantly affected (log2FC >0.585, FDR <0.05 from three biological replicates for each KD) by Eya3 or B55α KD. *B*, phosphoserine (pS) and phosphothreonine (pT) amino acid motifs found enriched in WT compared to Eya3 KD cells. *C*, pS and pT amino acid motifs found enriched in WT compared to B55α KD cells. The dataset comprised two distinct biological replicates in panels *B* and *C*. Each dot (.) in the motif indicated an amino acid of any identity. Eya, eyes absent; FDR, false discovery rate; KD, knockdown.

As an effort to evaluate whether Eya3 affects PP2A-B55α’s substrate preference, we knocked down Eya3 and B55α in 66cl4 triple-negative breast cancer cells and performed phosphoproteomic analyses after TiO2 enrichment [which enriches pThr and pSer instead of pTyr phosphopeptides (49)], in order to identify changes in phosphosites in response to each KD condition (Fig. S5). These analyses showed that both KDs had substantial phosphosite overlap (Fig. 6*A*). We further analyzed the top phosphosite motifs in the Eya3 and B55α KDs using PhosphoSite Plus as outlined in the Experimental procedures. We found that the motifs with the highest Z-scores were highly similar between Eya3 and B55α KDs (Fig. 6, *B* and *C*). These observations support the notion that PP2A-B55α mediates Eya3’s apparent phosphatase activity, and they suggest that Eya3 does not significantly affect PP2A-B55α’s substrate preference (otherwise we would expect the phosphosite motif of the Eya3 KD to be significantly different from that of the B55α KD). In particular, the top two phosphosite motifs of Eya3 and B55α are both pSP and pTP (pSer and pThr followed by Pro). This finding is consistent with previous studies showing that PP2A-B55α substrates tend to have Pro in the n+1 position of a pThr or pSer (48) and consistent with the fact that both T58 and S62 residues are followed by a Pro in Myc.

### PP2A-B55α dephosphorylates a substrate with a non-Pro residue following pThr more efficiently

**Figure 7:**
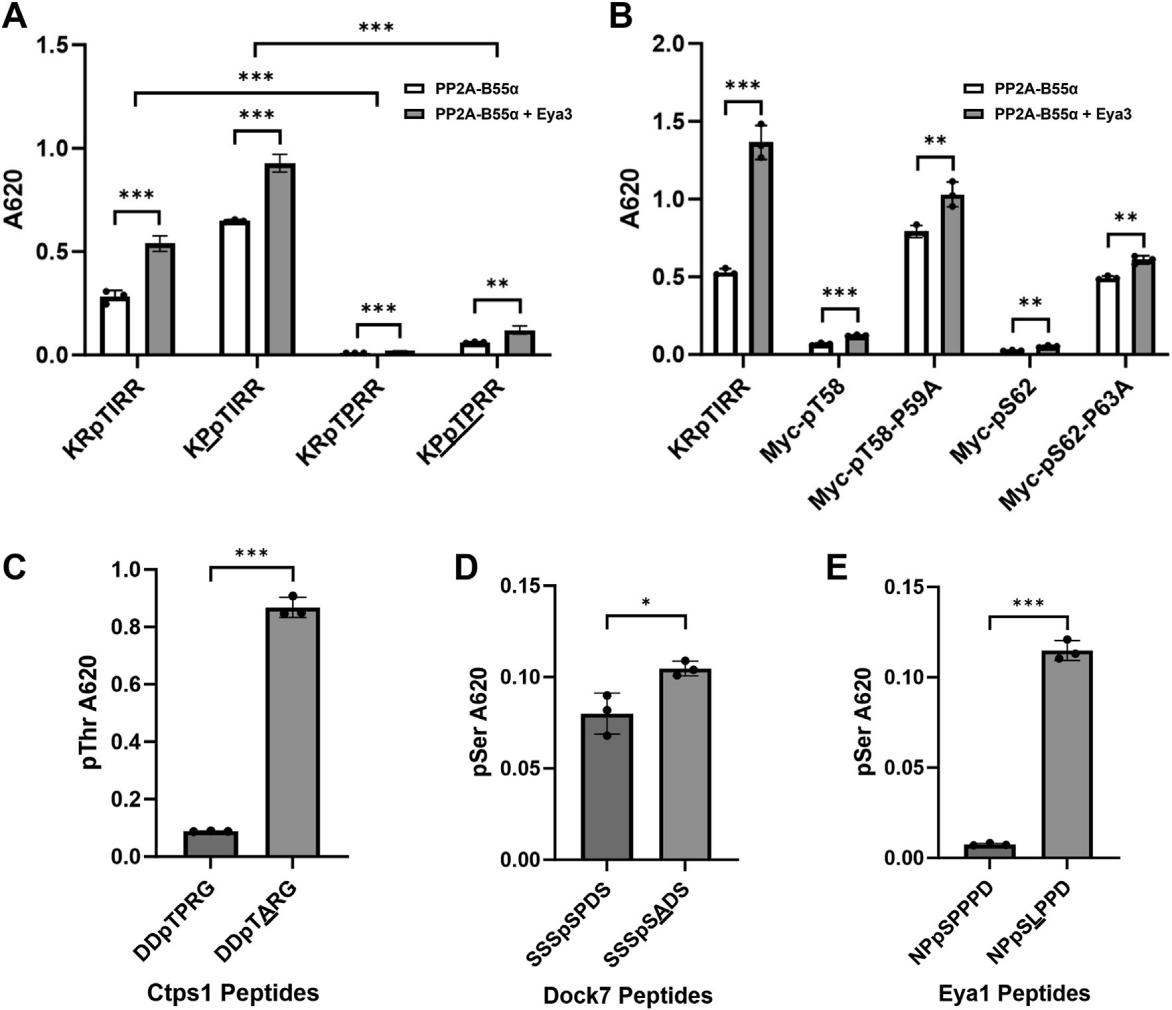
**PP2A-B55α prefers a non-Pro residue following phospho-Thr or Ser.***A*, malachite green assay showing that PP2A-B55α preferred Pro before pThr, not after pThr, on the generic substrate KRpTIRR. Residues different from the parental peptide are *underlined* in panels *A* and *C*–*E*. *B*, PP2A-B55α had much higher activity when Pro was mutated to Ala following pThr or pSer in the Myc peptide. Eya3 increased PP2A-B55α activity but did not change this preference. *C*, PP2A-B55α phosphatase activity with the DDpTPRG Ctps1 and DDpTARG mutant peptide substrates. *D*, PP2A-B55α phosphatase activity with the SSSpSPDS Dock7 and SSSpSADS mutant peptide substrates. *E*, PP2A-B55α phosphatase activity with the NPpSPPPD Eya1 and NPpSAPPD mutant peptide substrates. Absorbances were measured at 620 nm (*A*_620_) after incubating for 15 min in panel *A* or 30 min in panels *B*–*E*. Statistical analysis was done on three biological replicates using Fmax tests followed by two-tailed, homoscedastic *t* tests. In this figure, ∗∗∗*p* < 0.001, ∗∗*p* < 0.01, and ∗*p* < 0.05. Error bars represented the SD. Eya, eyes absent; PP2A, protein phosphatase 2A.

Given the enrichment of peptides containing a pTP or pSP motif in the phosphoproteomic analysis with Eya3 and B55α KD, we wondered if a Pro in the n+1 position of pSer or pThr would elicit higher phosphatase activity of PP2A-B55α compared to other amino acids in the n+1 position of pSer or pThr. To test this hypothesis, we first evaluated the PP2A-B55α phosphatase activity on pThr with Pro either before or after the pThr residue using a generic PP2A-B55α peptide substrate (KRpTIRR). To our surprise, Pro in the n+1 (but not the n-1) position of the pThr residue precipitously decreased phosphatase activity (Fig. 7*A*). Interestingly, both endogenous pT58 and pS62 in Myc contain Pro residues in the n+1 position. Not surprisingly, PP2A-B55α activity with the generic pThr peptide is significantly higher than that of the endogenous pT58 and pS62 peptides (Fig. 7*B*). Mutating Pro in the n+1 position of pT58 or pS62 to Ala significantly increased dephosphorylation by PP2A-B55α on Myc peptides (Fig. 7*B*). Furthermore, the addition of Eya3 to PP2A-B55α increased the dephosphorylation on all peptides analyzed but did not change the fact that mutating the Pro in the n+1 position of pT58 or pS62 dramatically increases the dephosphorylation by PP2A-B55α (Fig. 7, *A* and *B*). To evaluate the generalizability of this observation, we further tested a Ctps1 and a Dock7 phosphopeptide that were both affected by the Eya3 and B55α KDs in addition to a phosphopeptide from Eya1. These phosphopeptides were all dephosphorylated by the PP2A-B55α holoenzyme, and the dephosphorylation increased when the n+1 position was mutated from a Pro to Ala or Leu (Fig. 7, *C*–*E*).

### The Pro residue after pT58 is necessary for maintaining the appropriate Myc level in cells

**Figure 8:**
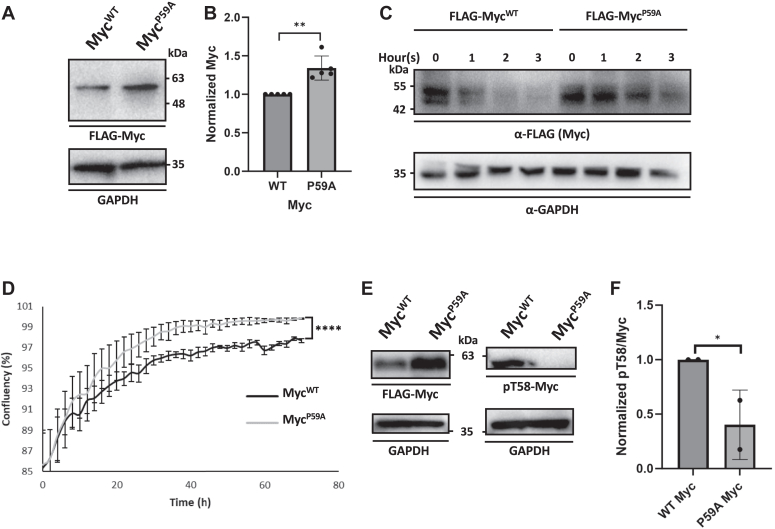
T**he Pro residue after pT58 is necessary for maintaining the appropriate Myc level in cells.***A*, FLAG-Myc^WT^ or mutant FLAG-Myc^P59A^ were transfected in HEK cells, and Myc level was probed in a Western blot using an α-FLAG antibody from whole cell lysate. *B*, quantification of the Myc^WT^ and Myc^P59A^ in panel *A*. *C*, mutant P59A increased Myc stability levels in HEK cells in a cycloheximide chase assay. *D*, HEK cells-transfected Myc^P59A^ had increased cell growth when compared to Myc^WT^. *E*, FLAG-Myc^WT^ or mutant FLAG-Myc^P59A^ were transfected in HEK cells. Myc and pT58-Myc levels were probed in Western blots using an α-FLAG antibody from whole cell lysate. *F*, quantification of pT58/Myc ratio in Myc^WT^ and Myc^P59A^. The dataset comprises five biological replicates in panel *B*, six biological replicates in panel *D*, and two biological replicates in panel *F*. Statistical analysis was done with an Fmax followed by a homoscedastic *t* test in panels *B* and *F*, and a repeated measures ANOVA in panel *D*. Error bars represented the SD. ∗*p* < 0.05, ∗∗*p* < 0.01, ∗∗∗∗*p* < 0.0001.

Myc^P59A^ is a hotspot mutation within the Myc phosphodegron in Burkitt’s lymphoma cancer patients (50). In the GENIE Cohort clinical trial, this mutation has also emerged as the second most common Myc mutation, spanning 45 patient cancer samples and 11 cancer types (51). Since pT58 is known to be a degradation signal on Myc, and the Myc^P59A^ mutation increases PP2A-B55α’s dephosphorylation of pT58, we speculated that the Myc^P59A^ mutation also increases Myc stability. To evaluate this hypothesis, we transfected HEK cells with either FLAG-Myc^WT^ or FLAG-Myc^P59A^ mutant and found that the Myc^P59A^ mutant consistently increased the Myc protein level in whole cell lysate (Fig. 8, *A* and *B*). We further showed that the Myc^P59A^ mutation decreased Myc degradation in a cycloheximide chase protein stability assay when compared to Myc^WT^ (Fig. 8*C*). We also showed that Myc^P59A^-transfected cells had an increased cell growth rate when compared to Myc^WT^ in an incucyte assay (Fig. 8*D*), likely due to the increased Myc level in the cell. Moreover, we found that Myc^P59A^ has increased dephosphorylation at pT58 compared to Myc^WT^ (Fig. 8, *E* and *F*), providing the molecular basis for the increased Myc stability (Fig. 8*C*).

## Discussion

In this work, we have demonstrated that the Eya3 CTD directly interacts with the purified Myc NTD, and knockdown of Eya3 reduces the Myc association with PP2A-B55α (Fig. 1). This and our previous data (12) together support a model where Eya3 directly interacts with PP2A-B55α through its NTD and with Myc through its CTD, recruiting PP2A-B55α to dephosphorylate Myc. We further demonstrated that Eya3 increases PP2A-B55α’s phosphatase activity with no effect on PP2A-B56α, consistent with our previous observation that Eya3 directly interacts with the B55 but not B56 subunit of PP2A (12). Further work will be necessary to elucidate the exact mechanism Eya3 uses to increase the phosphatase activity of PP2A-B55α, though several possibilities exist. First, the Eya3-binding site on B55α partially overlaps with the acidic substrate-binding site for pTau (Fig. 5, *A*–*D*). It is possible that Eya3 stabilizes substrate binding by B55α at the remaining open portion of the acidic binding pocket. It is also possible that Eya3 binding affects the conformation of B55α and consequently the catalytic C subunit.

We demonstrated that the entire NTD of Eya3 is intrinsically disordered and that Eya3 uses a segment spanning residues 53 to 90 to interact with B55α (Fig. 3, *A*–*E*). The minimal Eya3 segment that binds B55α can potentially be even shorter. We also demonstrated that Eya2 also uses a segment in its NTD (residues 32–54) to interact with B55α (Fig. 4, *D*–*F*), suggesting that this observation holds true for all Eya family members. The Eya2 segment (residues 32–54) does not exactly correspond to the Eya3 segment (residues 53–90) in sequence alignment, which is not surprising given that the NTDs of Eya proteins are highly divergent and a true alignment of these sequences is difficult (Fig. S4). The limited sequence similarities among the corresponding segment sequences in the NTD of different Eyas may help us identify specific residues critical for the interaction with B55α. Alternatively, the nature of the residues that interact with B55α may not be fixed, but instead the combined contribution from different residues in the NTD segment of different Eyas enables them to bind B55α.

This observation that Eya3 (and other Eyas) uses a disordered short segment in its NTD to interact with B55α also raises the intriguing possibility of inhibiting the interaction between Eya3 and PP2A using a peptide derived from Eya3 fused to a cell penetrating peptide (CPP). CPPs are short, positively charged peptides consisting of 10 to 30 amino acids that can enter the cell and nucleus through the cell and nuclear membranes, in most cell types, with its conjugated cargo (52, 53, 54, 55, 56, 57). Multiple CPPs are currently in clinical trials for the treatment of cancer and other diseases, including p28, OmoMyc, TRIP-PCI (PZ-128), and HEALOS (AM-111) (58, 59, 60, 61, 62). Given that the Eya3 and PP2A-B55α interaction plays a major role in promoting breast tumor growth and metastasis (12, 21), inhibiting this interaction may serve as a potential therapeutic approach for breast and other Eya and Myc-driven cancers.

Eya3 and B55α knockdown causes phosphorylation changes on proteins with highly similar sequence motifs represented by Ser or Thr followed by Pro (Fig. 6). The preference of PP2A-B55α for Ser or Thr followed by Pro has been previously documented (35). The fact that Eya3 affects the same sequence motifs supports our observation that Eya3’s apparent phosphatase activity is not intrinsic but comes from its direct interaction with PP2A-B55α (12). It also suggests that Eya3 does not affect the substrate preference of PP2A-B55α. Intriguingly, we found that the Pro residue in the n+1 position of pSer or pThr makes both a generic peptide and the Myc peptide a much poorer substrate for PP2A-B55α (Fig. 7). Consistent with this observation, a P59A mutant that occurs in Burkitt’s lymphoma and other cancers leads to significantly reduced level of pT58, increased stability of Myc, and consequently increased cell growth (Fig. 8). These observations beg the question why PP2A-B55α prefers Pro in the n+1 position of pThr and pSer despite the sequence being a much poorer substrate than pThr or pSer followed by non-Pro residues. One possibility is that the Pro residues in the n+1 position of pThr or pSer allows for the regulation of the dephosphorylation of these substrates by Pin1, similar to the regulation of PP2A-B56α–mediated pS62 dephosphorylation by Pin1 (63). Further experiments will be needed to verify this possibility and the role of Pin1 as a regulator of both PP2A-B55α and PP2A-B56α’s activities on distinct phosphosites on Myc.

## Experimental procedures

### Cloning

We engineered an N-terminal FLAG tag to human Eya3, human Eya2, and mouse Myc, as well as an N-terminal HA-tag to human B55α on a pEF-BOS vector (64) with the NEBuilder HiFi DNA Assembly kit from NEB. We also used the same kit to generate Myc, Eya2, and Eya3 truncations, as well as Myc and B55α mutations. We verified all constructs through Sanger sequencing.

### Protein purification

Overnight (ON) culture of either human GST-Aα (12), GST-B56α (Addgene #132635), mouse FLAG-Eya3, or mouse His-Myc_1–88_ plasmid-containing Rosetta cells were started in LB media supplemented with 50 μg/ml Ampicillin. ON culture was added to 50 μg/ml ampicillin-containing 2XYT media prewarmed to 37 °C. Bacteria was grown at 200 RPM and 37 °C until *A*_600_ 0.6. Cultures were then transferred to 25 °C and grown until the *A*_600_ was 0.9. The cultures were then induced with IPTG at 0.1 - 0.5 mM and incubated at 200 RPM and 25 °C for 18 h. Lysis buffer containing 50 mM Tris, pH 7.5, 250 mM NaCl, 5% glycerol, and 1 mM DTT was added to resuspend the pellet. Bacteria was lysed *via* sonication 5× 45 s/ea while on ice. NaCl was brought to 0.5 M for centrifugation by adding 5 M NaCl. The lysate was centrifuged at 18,000*g* for 40 min, and the supernatant was removed. Supernatant was loaded onto resin with gravity flow 2×. The protein-bound column was washed with 100 ml of lysis buffer. The bound protein was then cleaved ON at 4 °C with PreScission. Protein was eluted with 10 ml of 10 mM DTT-containing lysis buffer. The protein was then concentrated to 2 ml and loaded onto a prewashed S200 column. The peaks were collected and combined.

Purification of the other PP2A subunits was done using SF9 cells transduced with either His-Cα or His-B55α (12), and were lysed with sonication in lysis buffer containing 50 mM Tris (pH 7.5), 500 mM NaCl, 5% glycerol, 0.5% Triton X-100, 10 mM imidazole, and 1× PIC. Cell lysate was then centrifuged for 20 m at 15,000 RPM. Recharged and equilibrated Ni-NTA resin was then added to the lysate supernatant and rotated for 20 min at 4 °C. The beads were then washed 3× with lysis buffer, 2× with 20 mM imidazole, and 1× with 40 mM imidazole. The purified proteins were then eluted with 400 mM imidazole. Dialysis was then done to remove the imidazole. Purified proteins were verified with Coomassie-stained sodium dodecyl sulfate (SDS) gels.

### Malachite green phosphatase assays

Phospho-substrate (300–500 μM) was added to 100 to 500 nM phosphatase in 50 mM Tris (pH 7.5), 50 mM NaCl, 5 mM MgCl_2_, and 0.1 mM EDTA. Phosphatase reactions were conducted between 15 and 40 min at 37 °C in a 96-well plate. Reactions were then stopped by adding the malachite Green solution containing 0.01% tween, 0.034% malachite green in 10 mM ammonium molybdate, 1 N HCl, and 3.4% ethanol. The solution was then measured on a Molecular Devices plate spectrophotometer at 620 nm.

### Mammalian cell culture

HEK and 66cl4 cells were cultured in Dulbecco's modified Eagle's medium growth medium (Thermo Scientific) containing 10% FBS (Thermo Scientific) with 5% CO_2_ at 37 °C until 85% confluency was reached. Cells were collected and centrifuged at 1000 RPM at 4 °C for 10 min. Cell pellets were then stored at −20 °C. Cells were lysed after thawing with lysis buffer containing 1 mM dithiothreitol (DTT), 100 mM NaCl, 50 mM Tris (pH 7.5), and protease inhibitors (Thermo Scientific, A32963). All cell lines, including HEK and 66cl4, were validated by DNA fingerprinting (short tandem repeat/STR examination) in the University of Colorado Cancer Center Protein Production/MoAB/Tissue Culture Core, at the beginning of experiments. We also evaluated all lines for *Mycoplasma* using the e-Myco plus PCR Detection Kit (iNtRON).

### Mammalian cell transfection

DNA (10–20 μg) was added to 437 μl of pH 7.3 Tris-EDTA containing 10 mM Tris and 1 mM EDTA. Five hundred microliter of HEPES Buffered Saline consisting of 50 mM HEPES, 280 mM NaCl, 1.5 mM Na_2_HPO_4_, 10 mM KCl, and 12 mM dextrose is added in addition to 63 μl of 2 M CaCl_2_. This solution was then added dropwise to a 50 to 60% confluent mammalian cell culture. To do the B55α and Eya3 KDs, 66cl4 cells were transfected with shRNA construct, PVM I, II, and III. Forty microliter of 2 mg/ml polybrene (250×) was added to the 10 ml of media to enhance transduction. After 48 h, 5 μg/ml puromycin was used to start puromycin selection of positive cells. KDs were verified through Western blots of whole cell lysate. We have previously published detailed data on the efficacy and vectors of the 66cl4 Eya3 and B55α KDs (12).

### Co-immunoprecipitation

HEK cells were transfected with mouse FLAG-Eya3 CTD, wildtype human FLAG-Eya3, mutant human FLAG-Eya3, wildtype human HA-B55α, and mutant human HA-B55α plasmids. The cells were cultured, lysed, and transfected as mentioned in the Mammalian Cell Culture and Mammalian Cell Transfection sections above. FLAG gel (Sigma F2426) was resuspended with the vortexer and washed 3× with 1 ml of the lysis buffer. The cleaned FLAG gel was added to the cell lysate and rotated on a vertical rotator at 4 °C ON. The FLAG gel was washed 3× with 1 ml of the lysis buffer described above. Next, the co-IP product was eluted by incubating with 100 μl of 150 ng/μl FLAG peptide (Sigma F3290). The dissociated protein complex was immediately analyzed with a Western blot.

For B55α co-IP, cleaned Protein A/G beads were added to the 66cl4 cell lysate and rotated on a vertical rotator at 4 °C ON with α-B55α (Santa Cruz, sc-81606). The Protein A/G beads were washed 3× with 1 ml of the lysis buffer. Next, the co-IP product was eluted by boiling for 5 min with 1× SDS loading buffer. The dissociated protein complex was immediately analyzed with a Western blot.

### Western Blot

Cells were lysed with 1 ml of lysis buffer containing 40 mM HEPES pH 7.5, 150 mM NaCl, 0.5% Triton X-100, 10% glycerol, and 2 mM EDTA was used for each plate. The lysate was centrifuged with 12,000*g* 20 min at 4 °C. Cytoplasmic and nuclear fractions were collected with the NE-PER Nuclear and Cytoplasmic Extraction kit (Thermo Scientific, 78835). Samples were run on 12.5% SDS PAGE gels and transferred onto polyvinylidene fluoride (0.22 μM) membranes with 200 mAmps for 75 min. Polyvinylidene fluoride membranes were blocked in TBS-T with 5% milk at 25 °C for 1 h, and washed with 3× with TBST-T. The membrane was incubated at 4 °C ON with primary antibody. The membrane was then incubated at 25 °C for 1 h with the respective HRP-linked secondary antibodies: α-rabbit (Abcam ab6721), α-mouse (Thermo Scientific 31430), and α-rat, light-chain specific (CST 98164). The membrane was then imaged with high sensitivity chemiluminescence reagent (Bio-Rad) using the ChemiDoc XRS+ imaging system using Image Lab software (Bio-Rad).

Western blots were quantified with ImageJ (NIH) using inverted pixel density. For each protein, the same frame was used across all lanes. Relative quantification values were determined by dividing the protein of interest to the loading control in each lane. The normalization was then calculated by dividing each lane by the relative quantification in the control lane.

The following primary antibodies were used: α-FLAG (mouse, Sigma F3165), α-HA (rat, Roche 3F10), α-B55α (mouse, Santa Cruz sc-81606), α-EYA3 (rabbit, Proteintech 21196-1-AP), α-Myc pT58 (rabbit, ABM Y011034), α-β-actin (mouse, Santa Cruz sc-47778), α-Myc (rabbit, Abcam Y69), and α-GAPDH (mouse, GeneTex GT239). The specificity of the anti-Flag and anti-HA antibodies was validated by comparing cells transfected with or without Flag or HA-tagged plasmids. The other antibodies were validated by the corresponding commercial resources using the following approaches: Myc (immunohistochemistry (IHC), chromatin immunoprecipitation, Co-IP, gene knockout, Western); B55α (IHC and Western blot); pT58 Myc (IHC, Western blot, and phosphopeptide); and GAPDH (IHC, Western blot, and subcellular fractionation). We also validated the specificity of the B55α using recombinant proteins.

### Myc stability assay

HEK cells were transfected with either human Myc^WT^ or human Myc^P59A^. Forty-eight hours after transfection, cells were treated with 50 μg/μl of cycloheximide to pause translation. Samples were collected at 0, 1, 2, and 3 h to analyze Myc degradation levels by Western Blot.

### Incucyte assay

Cell proliferation was measured using the Sartorius Incucyte S3 Live-Cell Analysis Instrument to image HEK cells. Cells were analyzed using continuous high-definition phase contrast analysis of whole-well modules.

### Proteomics sample preparation, tandem mass tags labeling, and phosphoenrichment

Mouse 66cl4 breast cancer cells cultured with shRNA targeting either Eya3, PP2A-B55α, or a scrambled sequence were washed with PBS and immediately harvested with 5% (w/v) SDS, 10 mM tris(2-carboxyethylphosphine), 40 mM 2-chloroacetamide, and 50 mM Tris-HCl, pH 8.5 and boiled 10 min. Cell lysates were probe sonicated and stored at −80 °C. Extracted proteins were digested using the SP3 method (65). Briefly, 500 μg carboxylate-functionalized SpeedBeads (Cytiva Life Sciences) was added followed by the addition of acetonitrile to 80% (v/v) inducing binding to the beads. The beads were washed twice with 80% (v/v) ethanol and twice with 100% acetonitrile. Proteins were digested in 50 mM Tris-HCl, pH 8.5, with Lys-C/Trypsin (Promega) incubating at 37 °C shaking at 1000 rpm ON. Tryptic peptides were desalted using Waters HLB Oasis cartridge according to the manufacturer’s instructions and dried in a speedvac vacuum centrifuge and stored at −20 °C. Peptides were labeled with the tandem mass tags (TMT) 10-plex (Thermo Scientific) according to the manufacturer’s instructions. Briefly, peptides were suspended in 0.1 M triethylammonium bicarbonate, and each TMT label was added in acetonitrile and incubated for 1 h at ambient. TMT labeling reactions were quenched with the addition of hydroxylamine and incubated for 15 min at ambient then combined. The multiplexed samples were desalted using a Waters HLB Oasis cartridge according to the manufacturer’s instructions, dried in a speedvac vacuum centrifuge, and stored at −20 °C. A small portion of the multiplexed samples was set aside for proteome analyses, and the remaining was phosphoenriched using the High-Select TiO2 phosphoenrichment kit (Thermo Scientific). Samples were suspended in the binding/equilibration buffer, loaded onto the TiO2 column, washed, and eluted. The elution and the unretained flow through fractions were dried immediately in a speedvac vacuum centrifuge. The TiO2 elution was stored at −20 °C, while the dried unretained flow through fraction was subjected to a sequential phosphoenrichment with the High-Select Fe-NTA phosphoenrichment kit (Thermo Scientific). The dried TiO2 unretained fraction was suspended in the Fe-NTA binding/wash buffer, loaded onto the Fe-NTA column, washed and eluted. The TiO2 and the Fe-NTA elutions were combined and dried in a speedvac vacuum centrifuge. The proteome and phosphoenriched fractions were fractionated on a rpC18 column with high pH mobile phases (0.1% ammonium hydroxide) using a Waters M-class UPLC with a PDA detector and a custom fabricated 0.5 mm × 150 mm UChrom rpC18 1.8 μm 120 Å (nanolcms) column with a gradient from 2% to 40% acetonitrile (ACN) in 80 min for the proteome and from 2% to 25% ACN in 50 min for the phosphoproteome. Fractions were concatenated for a total of 24 proteome fractions and 12 phosphoproteome fractions. High pH fractions were immediately dried in a speedvac vacuum centrifuge and stored at −20 °C until LC/MS analysis.

### Mass spectrometry and data analyses

High pH-fractionated TMT-labeled peptides were suspended in 3% (v/v) ACN and 0.1% (v/v) trifluoroacetic acid and directly injected onto a reversed-phase CSH rpC18 1.7 μm, 130 Å, 75 mm × 250 mm M-class column (Waters), using an Ultimate 3000 nanoUPLC (Thermo Scientific). For the proteome analyses, peptides were eluted at 300 nl/min with a gradient from 2% to 20% ACN, 0.1% (v/v) formic acid in 40 min then to 40% ACN in 5 min and detected using a Q-Exactive HF-X mass spectrometer (Thermo Scientific). For the phosphoproteome analyses, peptides were eluted at 300 nl/min with a gradient from 2% to 20% ACN, 0.1% (v/v) formic acid in 120 min then to 40% ACN in 5 min and detected using a Q-Exactive HF-X mass spectrometer (Thermo Scientific). Precursor mass spectra (MS1) were acquired at a resolution of 120,000 from 380 to 1580 m/z with an automatic gain control target of 3E6 and a maximum injection time of 50 ms. Precursor peptide ion isolation width for MS2 fragment scans was 0.7 m/z with a 0.2 m/z isolation offset, and the top 12 most intense ions were sequenced. All MS2 spectra were acquired at a resolution of 60,000 with higher energy collision dissociation at 30% normalized collision energy. An automatic gain control target of 1E5 and 100 ms maximum injection time was used. Dynamic exclusion was set for 25 s with a mass tolerance of ±10 ppm. Rawfiles were searched against the Uniprot *Mus musculus* database UP000000589 downloaded 11/13/2020 using MaxQuant v1.6.14.0. Cysteine carbamidomethylation and the TMT label were considered fixed modifications, while methionine oxidation, protein N-terminal acetylation and phosphorylation at serine, threonine, or tyrosine were searched as variable modifications. All peptide and protein identifications were thresholded at a 1% false discovery rate (FDR). Proteome data were normalized using the cyclic loess method, then differential protein abundances were calculated using Limma (Bioconductor.org) to generate a linear model for fold-change estimation and standard error and then an Empirical Bayes implementation of an independent *t* test, where the null distribution is inferred from the global data, and treatment distribution is Bayesian updated. FDR (q-values) was then generated using the Benjamini–Hochberg Limma interpretation. Phosphoproteomics data were processed with Perseus 2.0.3.0, using a Welch’s *t* test with a Benjamini–Hochberg FDR of 0.05.

We performed the following analyses to identify phosphosite motifs in Eya3 and B55α KDs. Each experimental group (two individual KDs for Eya3 and B55α each) was sorted from highest to lowest phosphoenrichment with KD using the Log2FC between the KD and SCR control to select for possible phosphatase targets. Data were filtered to include only phosphoenrichment in the KD group (relative to the SCR control) with log fold changes >1 and *p*-values <0.05. Filtered sequences were truncated to be 15 amino acids long with the phosphorylated residue as the center amino acid (included seven residues on either side of phosphorylated residue). These processed sequences in ranked order were then used as inputs into the Motif and Logo Analysis Tool from PhosphoSite Plus (https://www.phosphosite.org/staticMotifAnalysis) (66). Output lists were sorted by outputted Z-score (highest to lowest) which represents the magnitude of change from the mean in standard deviations.

### Circular dichroism

Recombinant mouse Eya3 NTD (residues 1–287) (3 mM) was reconstituted in 10 mM PBS and analyzed from 5 to 95 °C in increments of 5 °C. Molar ellipticity was measured at 0.1 nm intervals between 190 and 260 nm using the JASCO 815 CD Spectrometer.

## Data availability

All data are contained within the manuscript. Further information regarding the experiments may be found upon request to the corresponding author.

## Supporting information

This article contains supporting information (46).

## Conflict of interest

The authors declare no conflict of interest with the contents of this article.
