# Supplementary material for: Biochemical characterization of the Eya and PP2A-B55α interaction
Source: J Biol Chem. 2024 May 23;300(7):107408. doi: 10.1016/j.jbc.2024.107408 (PMC11328874; doi:10.1016/j.jbc.2024.107408)
Supplement: Supplemental Figure and Table legends [file mmc2.pdf]

**Supplementary figure legends**

**Fig. S1.** Coomassie blue stains of purified PP2A subunits GST-Aα, His-B55α, His-B56α, and His-Cα.

**Fig. S2.** Secondary structure prediction of Eya family proteins using Jpred4 (44) indicate that the NTD of Eya 3 (a), Eya1 (b), Eya2 (c), and Eya4 (d) are all intrinsically disordered by. -, E, and H represent disordered, β-strand, and α-helical region, respectively. Black and gray represent the NTD and CTD of Eya proteins.

**Fig. S3.** Eya3 deletions do not affect its cellular localization. WT FLAG-Eya3 and FLAG-Eya3 with deletions of residues Δ53-65, Δ66-78, and Δ79-90 were transfected into HEK cells, separated into cytoplasmic and nuclear fractions, and probed in Western blots with α-FLAG-Eya3, α-GAPDH, and α-HDAC1 antibodies. The Western blots displayed were representative of two biological replicates.

**Fig. S4.** Eya family NTDs (separated from the CTD by a vertical line) are more divergent than their CTDs as shown by sequencing alignment using COBALT alignment (46). Gray residues have deletions in one or more of the alignments. Blue residues have at least one mismatch amongst the Eya family alignment. Red residues are identical across the Eya family alignment. The red line indicates Eya3 residues 54-120 and the corresponding residues in other Eyas in the sequence alignment. The Eya1-4 protein sequences used in the alignment are from GenBank ID AAI21799.1, CAA71310.1, CAA71311.1, and CAA76636.1, respectively.

**Fig. S5.** 66cl4 cells with Eya3 (a) or B55a (b) KD (shown by Western Blot) were used for phosphoproteomic analyses.

**Supplementary Table 1.** Overlapping phosphopeptides between Eya3 and B55α KD. The pTP and pTS motifs were highlighted in blue and red font, respectively.
