# Supplementary material for: Biochemical characterization of the Eya and PP2A-B55α interaction
Source: J Biol Chem. 2024 May 23;300(7):107408. doi: 10.1016/j.jbc.2024.107408 (PMC11328874; doi:10.1016/j.jbc.2024.107408)
Supplement: Supplemental Figures S1–S5 [file mmc3.pdf]

Figure S1

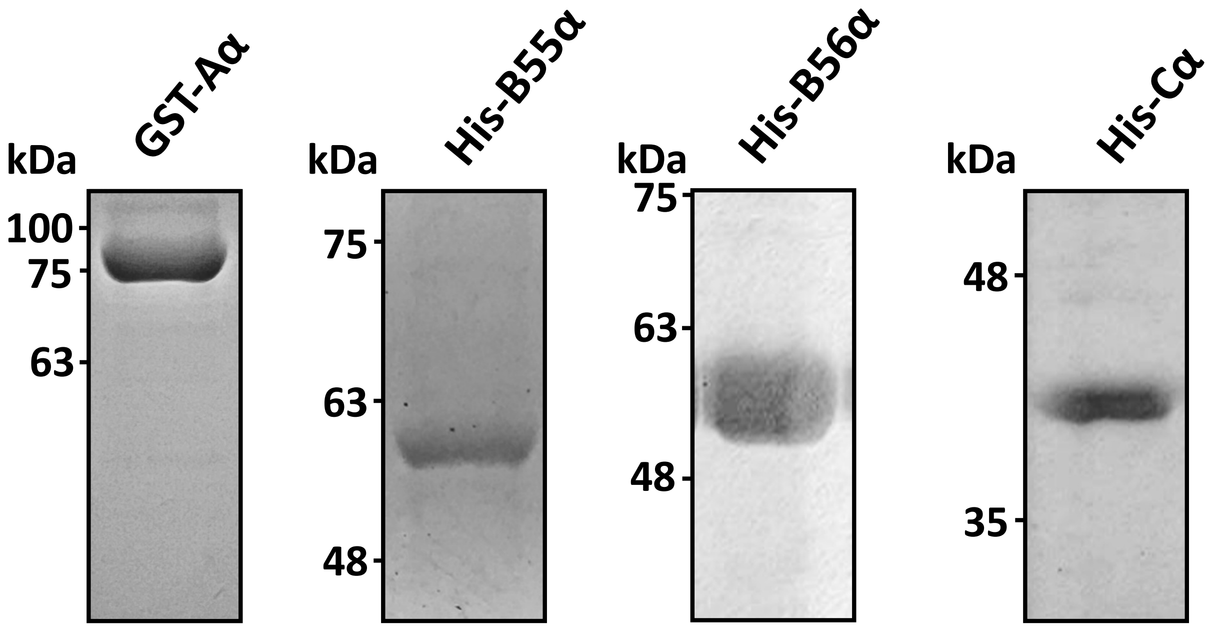
kDa
GST-Aα
100
75
63
kDa
His-B55α
75
63
48
kDa
His-B56α
75
63
48
kDa
His-Cα
48
35

Figure S2

A

Eya3

```
  1 MEEEQDLPEQPVKKAKMQESGEQTISQVSNPDVSDQKPETSSLASNLPMSEEIMTCTDYIPRSSNDYTSQMYSAKPY
    -----------------------------------------------------------------------------
 78 AHILSVPVSETAYPGQTQYQTLQQTQPDAVYPQATQTYGLPPFGALWPGMKPESGLIQTPSPSQHSVLTCTTGLTTS
    -----------------------------------------------------------------------------
155 QPSPAHYSYPIQASSTNASLISTSSTIANIPAAAVASISNQDYPTYTILGQNQYQACYPSSSFGVTGQTNSDAESTT
    -----------------------------------------------------------------------------
232 LAATTYQSEKPSVMAPAPAAQKLSSGDPSTSPSLSQTTPSKDTDDQSRKNMNSKNRGKKKADATSSQDSELERVFLW
    -----------------------------------------------------------------------------
309 DLDETIIIFHSLLTGSYAQKYGKDPTVVIGSGLTMEKMIFEVADTHLFSNDLKECDQVHVEDVAPNDKGQNLNNYSF
    -----HEE----------------HHHHHHHHHHHHHHHHHHHHH--H-----------------------------
386 STNGFSGSGGSGSHGSSVGVQGGVDWMRKLAFRYRKVREIYDKHKSNVGGLLSPQRKEALQKLKAEIEVLTNSWLGT
    ------------------------HHHHHHHHHHHHHHHHHHHH---------HHHHHHHHHHHHHHHHHHHHHHHH
463 ALKSLLLIQSKKNCVNVLITTTQLLPALAKVLLYGLGKIFPIENIYSATKIGKESCFERIVTSLGKKLTYVVIGDGR
    HHHHHHHHH-----EEEEEE---HHHHHHHHHH-------------------HHHHHHHHHHH----EEEEEEE---
540 DEEIAAKQHNMPFWRITNHGDLVSLHQALELDFL
    HHHHHHHH-----EEE---HHHHHHHHHHH----
```

Figure S2

B

Eya1

```
1   MFPSNAWAFYFLSFLTNSRPYPHILPTPSSQTMAAYGQTQFTTGMQQATAYATYPQPGQPYGISSYGALWAGIKTEG
    -----------------------------------------------------------------------------
78  GLSQSQSPGQTGFLSYGTSFSTPQPGQAPYSYQMQGSSFTTSSGIYTGNNSLTNSSGFNSSQQDYPSYPSFGQGQYA
    -----------------------------------------------------------------------------
155 QYYNSSPYPAHYMTSSNTSPTTPSTNATYQLQEPPSGITSQAVTDPTAEYSTIHSPSTPIKDSDSDRLRRGSDGKSR
    -----------------------------------------------------------------------------
232 GRGRRNNNPSPPPDSDLERVFIWDLDETIIVFHSLLTGSYANRYGRDPPTSVSLGLRMEEMIFNLADTHLFFNDLEE
    ------------------------------------------------HHHHHHHHHHHHHHHHHH-----------
309 CDQVHIDDVSSDDNGQDLSTYNFGTDGFPAAATSANLCLATGVRGGVDWMRKLAFRYRRVKEIYNTYKNNVGGLLGP
    --------EE------------------------------------HHHHHHHHHHHHHHHHHHHH----------H
386 AKREAWLQLRAEIEALTDSWLTLALKALSLIHSRTNCVNILVTTTQLIPALAKVLLYGLGIVFPIENIYSATKIGKE
    HHHHHHHHHHHHHHHHHHHHHHHHHHHHHHHH-----EEEEEEHHHHHHHHHHHHHH----------EE-------H
463 SCFERIIQRFGRKVVYVVIGDGVEEEQGAKKHAMPFWRISSHSDLMALHHALELEYL
    HHHHHHHHH----EEEEEEE---HHHHHHHH-----EEE----HHHHHHHHH-----
```

Figure S2

C

Eya2

```
1   MVELVISPSLTVNSDCLDKLKFNRADAAVWTLSDRQGITKSAPLRVSQLFSRSCPRVLPRQPSTAMAAYGQTQYSAG
    -----------------------------------------------------------------------------
78  IQQATPYTAYPPPAQAYGIPSYSIKTEDSLNHSPGQSGFLSYGSSFSTSPTGQSPYTYQMHGTTGFYQGGNGLGNAA
    -----------------------------------------------------------------------------
155 GFGSVHQDYPSYPGFPQSQYPQYYGSSYNPPYVPASSICPSPLSTSTYVLQEASHNVPNQSSESLAGEYNTHNGPST
    -----------------------------------------------------------------------------
232 PAKEGDTDRPHRASDGKLRGRSKRSSDPSPAGDNEIERVFVWDLDETIIIFHSLLTGTFASRYGKDTTTSVRIGLMM
    -------------------------------------EEEE-------------------------HHHHHHHHHHH
309 EEMIFNLADTHLFFNDLEDCDQIHVDDVSSDDNGQDLSTYNFSADGFHSSAPGANLCLGSGVHGGVDWMRKLAFRYR
    HHHHHHHHHH--------------E----------------------------------------HHHHHHHHHHHH
386 RVKEMYNTYKNNVGGLIGTPKRETWLQLRAELEALTDLWLTHSLKALNLINSRPNCVNVLVTTTQLIPALAKVLLYG
    HHHHHHHHH----------HHHHHHHHHHHHHHHHHHHHHHHHHHHHHHHH----EEEEEEE---HHHHHHHHHHH-
463 LGSVFPIENIYSATKTGKESCFERIMQRFGRKAVYVVIGDGVEEEQGAKKHNMPFWRISCHADLEALRHALELEYL
    -----------------HHHHHHHHHHH----EEEEEEE----HHHHHH-----EEEE----HHHHHHHHH-----
```

Figure S2

D

Eya4

```
1   MEDSQDLNEQSVKKTCTESDVSQSQNSRSMEMQDLASPHTLVGGGDTPGSSKLEKSNLSSTSVTTNGTGGENMTVLN
    -----------------------------------------------------------------------------
78  IADWLLSCNTPSSATMSLLAVKTEPLNSSETTATTGDGALDTFTGSVITSSGYSPRSAHQYSPQLYPSKPYPHILST
    -----------------------------------------------------------------------------
155 PAAQTMSAYAGQTQYSGMQQPAVYTAYSQTGQPYSLPTYDLGVMLPAIKTESGLSQTQSPLQSGCLSYSPGFSTPQP
    -----------------------------------------------------------------------------
232 GQTPYSYQMPGSSFAPSSTIYANNSVSNSTNFSGSQQDYPSYTAFSQNQYAQYYSASTYGAYMTSNNTADGTPSSTS
    -----------------------------------------------------------------------------
309 TYQLQESLPGLTNQPGEFDTMQSPSTPIKDLDERTCRSSGSKSRGRGRKNNPSPPPDSDLERVFVWDLDETIIVFHS
    ----------------------------------------------------------------------EEEE---
386 LLTGSYAQKYGKDPPMAVTLGLRMEEMIFNLADTHLFFNDLEECDQVHIDDVSSDDNGQDLSTYSFATDGFHAAASS
    -----------------HHHHHHHHHHHHHH--------------EEEEEEEEE-----------------------
463 ANLCLPTGVRGGVDWMRKLAFRYRRVKELYNTYKNNVGGLLGPAKRDAWLQLRAEIEGLTDSWLTNALKSLSIISTR
    ------------HHHHHHHHHHHHHHHHHHHHHHH----------HHHHHHHHHHHHHHHHHHHHHHHHHHHHHH—
540 SNCINVLVTTTQLIPALAKVLLYSLGGAFPIENIYSATKIGKESCFERIMQRFGRKVVYVVIGDGVEEEQAAKKHNM
    --EEEEEEE----HHHHHHHHHHH-----------------HHHHHHHHHHH----EEEEEEE----HHHHHHH---
617 PFWRISSHSDLLALHQALELEYL
    -EEEE---HHHHHHHHHHH----
```

Figure S3

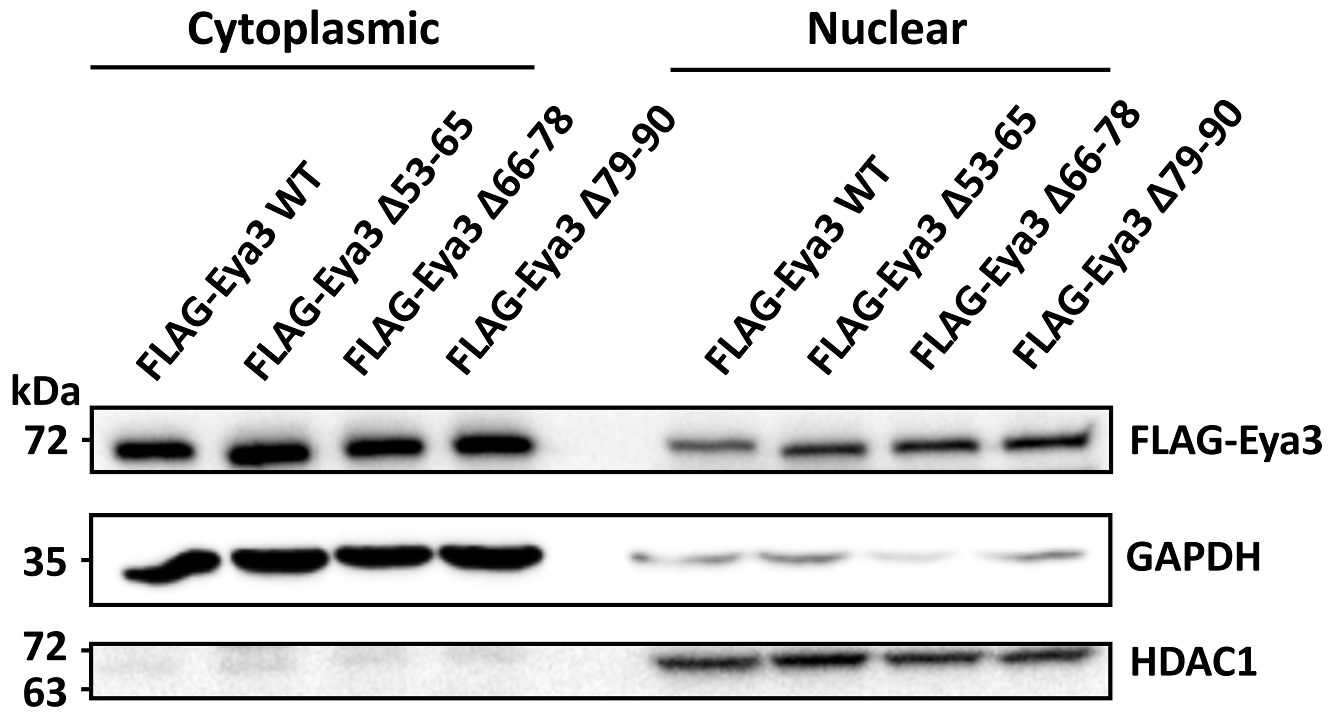
Cytoplasmic
Nuclear
FLAG-Eya3 WT
FLAG-Eya3 Δ53-65
FLAG-Eya3 Δ66-78
FLAG-Eya3 Δ79-90
FLAG-Eya3 WT
FLAG-Eya3 Δ53-65
FLAG-Eya3 Δ66-78
FLAG-Eya3 Δ79-90
kDa
72
FLAG-Eya3
35
GAPDH
72
63
HDAC1

Figure S4

```
EYA1          --------------------------------------------------------------------------------
EYA2          --------------------------------------------------------------------------------
EYA3   1      MEEEQDLPEQPVKK-------AKMQESGEQTISQVSNPDV--------SDQKPETSSLAS--------------------   45
EYA4   1      MEDSQDLNEQSVKKtctesdvSQSQNSRSMEMQDLASPHTlvgggdtpGSSKLEKSNLSStsvttngtggenmtvlniad   80

EYA1   1      -------------------------------------------MFPSNAW----AFYFLSFLTNSRPYPHILPTPSSQTM   33
EYA2   1      ---MVELVISPSLTVNSDCLDKLKFNRA-----DAAVWT-----LSDRQGITKSAPLRVSQLF-SRSCPRVLPRQPSTAM   66
EYA3   46     -----NLPMSE-------------------------------EIMTCTDYIPRSSNDYTSQMYSAKPYAHILSVPVSET-   88
EYA4   81     wllSCNTPSSATMSLLAVKTEPLNSSETtattgDGALDTftgSVITSSGYSPRSAHQYSPQLYPSKPYPHILSTPAAQTM   160

EYA1   34     AAY-GQTQFTTGMQQATAYATYPQPGQPYGISSY--GALWAGIKTEGGLSQSQSPGQTGFLSYGTSFSTPQPGQAPYSYQ   110
EYA2   67     AAY-GQTQYSAGIQQATPYTAYPPPAQAYGIPSY-------SIKTEDSL--NHSPGQSGFLSYGSSFSTSPTGQSPYTYQ   136
EYA3   89     -AYPGQTQYQ-TLQQTQPDAVYPQATQTYGLPPF--GALWPGMKPESGLIQTPSPSQHSVLTCTTGLTTSQPSPAHYSYP   164
EYA4   161    SAYAGQTQYS-GMQQPAVYTAYSQTGQPYSLPTYdlGVMLPAIKTESGLSQTQSPLQSGCLSYSPGFSTPQPGQTPYSYQ   239

EYA1   111    MQGSSFTTSSGIYTGNNSLTN---SSGFNSSQQDYPSYPSFGQGQYAQYYNSSPYPAHYMTSSNTSPTTPSTNATYQLQE   187
EYA2   137    MHGTT-----GFYQGGNGLGN---AAGFGSVHQDYPSYPGFPQSQYPQYYGSS-YNPPYVPASSICPS-PLSTSTYVLQE   206
EYA3   165    IQASS--TNASLISTSSTIANipaAAVASISNQDYPTYTILGQNQYQACYPSSSFGVTGQTNSD-AESTTLAATTYQSEK   241
EYA4   240    MPGSSFAPSSTIYA-NNSVSN---STNFSGSQQDYPSYTAFSQNQYAQYYSASTYGA-YMTSNNTADGTPSSTSTYQLQE   314

EYA1   188    PPSGITSQAVTDPTAEYSTIHSPS----TPIKDSDSDRLRRGSDGKSRGRGRRNNNPSPPPDSDL|ERVFIWDLDETIIVF   263
EYA2   207    ASHNVPNQSSESLAGEYNTHNGPS----TPAKEGDTDRPHRASDGKLRGRSKRSSDPSPAGDNEI|ERVFVWDLDETIIIF   282
EYA3   242    PSVMAPAPAAQKL-SSGDPSTSPSlsqtTPSKDTD-DQSRKNMNSKNRGKKKADA--TSSQDSEL|ERVFLWDLDETIIIF   317
EYA4   315    SLPGLTNQP-----GEFDTMQSPS----TPIKDLD-ERTCRSSGSKSRGRGRKNN-PSPPPDSDL|ERVFVWDLDETIIVF   383

EYA1   264    HSLLTGSYANRYGRDPPTSVSLGLRMEEMIFNLADTHLFFNDLEECDQVHIDDVSSDDNGQDLSTYNFGTDGFPAAATSA   343
EYA2   283    HSLLTGTFASRYGKDTTTSVRIGLMMEEMIFNLADTHLFFNDLEDCDQIHVDDVSSDDNGQDLSTYNFSADGFHSSAPGA   362
EYA3   318    HSLLTGSYAQKYGKDPTVVIGSGLTMEKMIFEVADTHLFSNDLKECDQVHVEDVAPNDKGQNLNNYSFSTNGFSGSGGSG   397
EYA4   384    HSLLTGSYAQKYGKDPPMAVTLGLRMEEMIFNLADTHLFFNDLEECDQVHIDDVSSDDNGQDLSTYSFATDGFHAAASSA   463

EYA1   344    NLCLATGVRGGVDWMRKLAFRYRRVKEIYNTYKNNVGGLLGPAKREAWLQLRAEIEALTDSWLTLALKALSLIHSRTNCV   423
EYA2   363    NLCLGSGVHGGVDWMRKLAFRYRRVKEMYNTYKNNVGGLIGTPKRETWLQLRAELEALTDLWLTHSLKALNLINSRPNCV   442
EYA3   398    SHGSSVGVQGGVDWMRKLAFRYRKVREIYDKHKSNVGGLLSPQRKEALQKLKAEIEVLTNSWLGTALKSLLLIQSKKNCV   477
EYA4   464    NLCLPTGVRGGVDWMRKLAFRYRRVKELYNTYKNNVGGLLGPAKRDAWLQLRAEIEGLTDSWLTNALKSLSIISTRSNCI   543

EYA1   424    NILVTTTQLIPALAKVLLYGLGIVFPIENIYSATKIGKESCFERIIQRFGRKVVYVVIGDGVEEEQGAKKHAMPFWRISS   503
EYA2   443    NVLVTTTQLIPALAKVLLYGLGSVFPIENIYSATKTGKESCFERIMQRFGRKAVYVVIGDGVEEEQGAKKHNMPFWRISC   522
EYA3   478    NVLITTTQLLPALAKVLLYGLGKIFPIENIYSATKIGKESCFERIVTSLGKKLTYVVIGDGRDEEIAAKQHNMPFWRITN   557
EYA4   544    NVLVTTTQLIPALAKVLLYSLGGAFPIENIYSATKIGKESCFERIMQRFGRKVVYVVIGDGVEEEQAAKKHNMPFWRISS   623

EYA1   504    HSDLMALHHALELEYL   519
EYA2   523    HADLEALRHALELEYL   538
EYA3   558    HGDLVSLHQALELDFL   573
EYA4   624    HSDLLALHQALELEYL   639
```

Figure S5

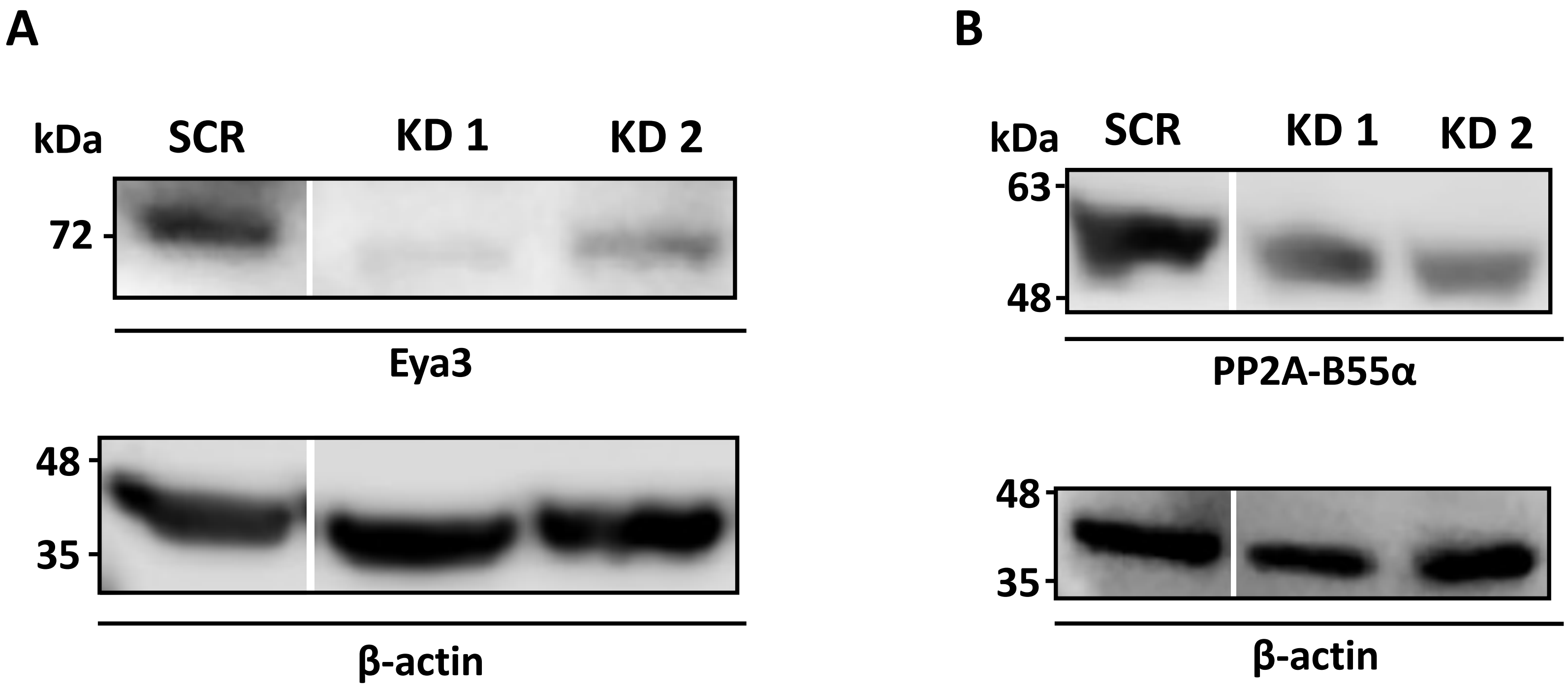

Eya3 KD in 66cl4 cells

B55α KD in 66cl4 cells
